# The Rhizosphere Microbiomes of Five Species of Coffee Trees

**DOI:** 10.1128/spectrum.00444-22

**Published:** 2022-03-15

**Authors:** Leandro Pio de Sousa, Oliveiro Guerreiro-Filho, Jorge Maurício Costa Mondego

**Affiliations:** a Department of Genetics, Evolution, Microbiology and Immunology, Institute of Biology, State University of Campinas, Campinas, Brazil; b Centro de Café Alcides Carvalho, Instituto Agronômico (IAC), Campinas, São Paulo, Brazil; c Centro de Pesquisa e Desenvolvimento de Recursos Genéticos Vegetais, Instituto Agronômico, Campinas, São Paulo, Brazil; USDA—San Joaquin Valley Agricultural Sciences Center

**Keywords:** *Coffea*, microbiome, rhizosphere-inhabiting microbes

## Abstract

Coffee is one of the most important commodities in the global market. Of the 130 species of *Coffea*, only Coffea arabica and Coffea canephora are actually cultivated on a large scale. Despite the economic and social importance of coffee, little research has been done on the coffee tree microbiome. To assess the structure and function of the rhizosphere microbiome, we performed a deep shotgun metagenomic sequencing of the rhizospheres of five different species, C. arabica, C. canephora, Coffea stenophylla, Coffea racemosa, and Coffea liberica. Our findings indicated that C. arabica and C. stenophylla have different microbiomes, while no differences were detected between the other *Coffea* species. The core rhizosphere microbiome comprises genera such as Streptomyces, Mycobacterium, Bradyrhizobium, Burkholderia, Sphingomonas, Penicillium, Trichoderma, and Rhizophagus, several of which are potential plant-beneficial microbes. Streptomyces and mycorrhizal fungi dominate the microbial communities. The concentration of sucrose in the rhizosphere seems to influence fungal communities, and the concentration of caffeine/theobromine has little effect on the microbiome. We also detected a possible relationship between drought tolerance in *Coffea* and known growth-promoting microorganisms. The results provide important information to guide future studies of the coffee tree microbiome to improve plant production and health.

**IMPORTANCE** The microbiome has been identified as a fundamental factor for the maintenance of plant health, helping plants to fight diseases and the deleterious effects of abiotic stresses. Despite this, in-depth studies of the microbiome have been limited to a few species, generally with a short life cycle, and perennial species have mostly been neglected. The coffee tree microbiome, on the other hand, has gained interest in recent years as *Coffea* trees are perennial tropical species of enormous importance, especially for developing countries. A better understanding of the microorganisms associated with coffee trees can help to mitigate the deleterious effects of climate change on the crop, improving plant health and making the system more sustainable.

## INTRODUCTION

Soil habitats are traditionally divided into four groups according to physical proximity to the plant host: bulk soil (the portion of soil furthest from the root), the rhizosphere (the thin layer of soil surrounding roots), the rhizoplane (the root surface colonized by microorganisms), and the endosphere (internal root tissues) (1). Until now, the soil-associated microbiomes of several food crops have been studied, for example, rice (2), maize (3), soybeans (4), grapevines (5), citrus (6), and wheat (7). The investigation of the microbiomes has shown that each plant contains a core group of microorganisms and that the microbial community structures can vary according to intrinsic characteristics of the host (8) and environmental characteristics like altitude (9), type of soil (10), and rainfall (11). It has long been known that this microbiota can directly affect plant health, promoting growth, suppressing pathogens, and reducing the deleterious effects of salinity, water stress, and xenobiotics (12). Thus, in seeking to take advantage of this phenomenon to benefit agriculture, great effort has been expended to understand how the microbiota affects the host’s health and its vigor in the face of several stresses (12).

Coffee is one of the most important commodities in the global market and a source of subsistence for more than 125 million people in Latin America, Africa, and Asia (13). Of the 130 species of the *Coffea* genus, Coffea arabica and Coffea canephora are the species most widely planted, comprising 70 and 30% of global production, respectively (14). However, some wild species, such as Coffea racemosa, Coffea liberica, and Coffea stenophylla, are planted on a small scale with regional importance. Besides, they provide genes for the improvement of Coffea arabica through crossing, aiming at the development of varieties that are more tolerant to abiotic and biotic stresses (15–17). Recently, some of these wild *Coffea* species also have gained a lot of interest for their arabica-like flavor and for growing at high temperatures, which C. arabica does not tolerate (18). These characteristics make them a part of possible responses to the climate crisis, which could have a major impact on coffee growing (17). Given the economic interest in coffee growing, coffee tree genomes, especially that of C. arabica, have been extensively studied, allowing a better understanding of genetic control of plant-microorganism interactions (19–23). However, there are still areas with important gaps regarding the study of these interactions. One of these areas is microbiome research, an area that has gained more interest recently but is still incipient (24).

In view of the limited literature on the *Coffea* microbiome, in this study, we collected rhizosphere samples from five species of coffee trees (the traditional C. arabica and C. canephora and the wild species C. stenophylla, C. racemosa, and C. liberica; see the supplemental material for more information about the characteristics of each species) present in the coffee collection of the Instituto Agronômico de Campinas (IAC), Brazil, and we conducted deep shotgun metagenomic sequencing of the rhizosphere microbiota. Here, we present a comprehensive taxonomic and functional analysis of the microbiomes of these different but related coffee species.

## RESULTS

### General information.

To assess the structure and function of the *Coffea* rhizosphere microbiome, we performed deep shotgun metagenomic sequencing. Ninety-four giga–base pairs (Gbp) of shotgun metagenomic sequences were generated. After the removal of *Coffea* sequences (less than 0.001% of the clean reads), assembly was performed using metaSPAdes. On average, 40% of the reads were used for metagenomic contig construction (Table S1 in the supplemental material). About 20 million nonredundant genes were then clustered. Prokaryotes represented 99% of the total annotated metagenes (Table S2). The rest of the metagenes were annotated as eukaryotic, including fungi, protozoa, and algae. Viral genes represented less than 0.02% of the annotated metagenes. The dominant prokaryotic phyla found included *Proteobacteria* and *Actinobacteria*, with more than 70% of abundance (Fig. S1a), similar to the percentages found in the rhizospheres of other species (2–7), while in the fungal community, *Ascomycota* and *Mucoromycota* prevailed with more than 80% of the abundance (Fig. S1b).

### Taxonomic composition.

**FIG 1:**
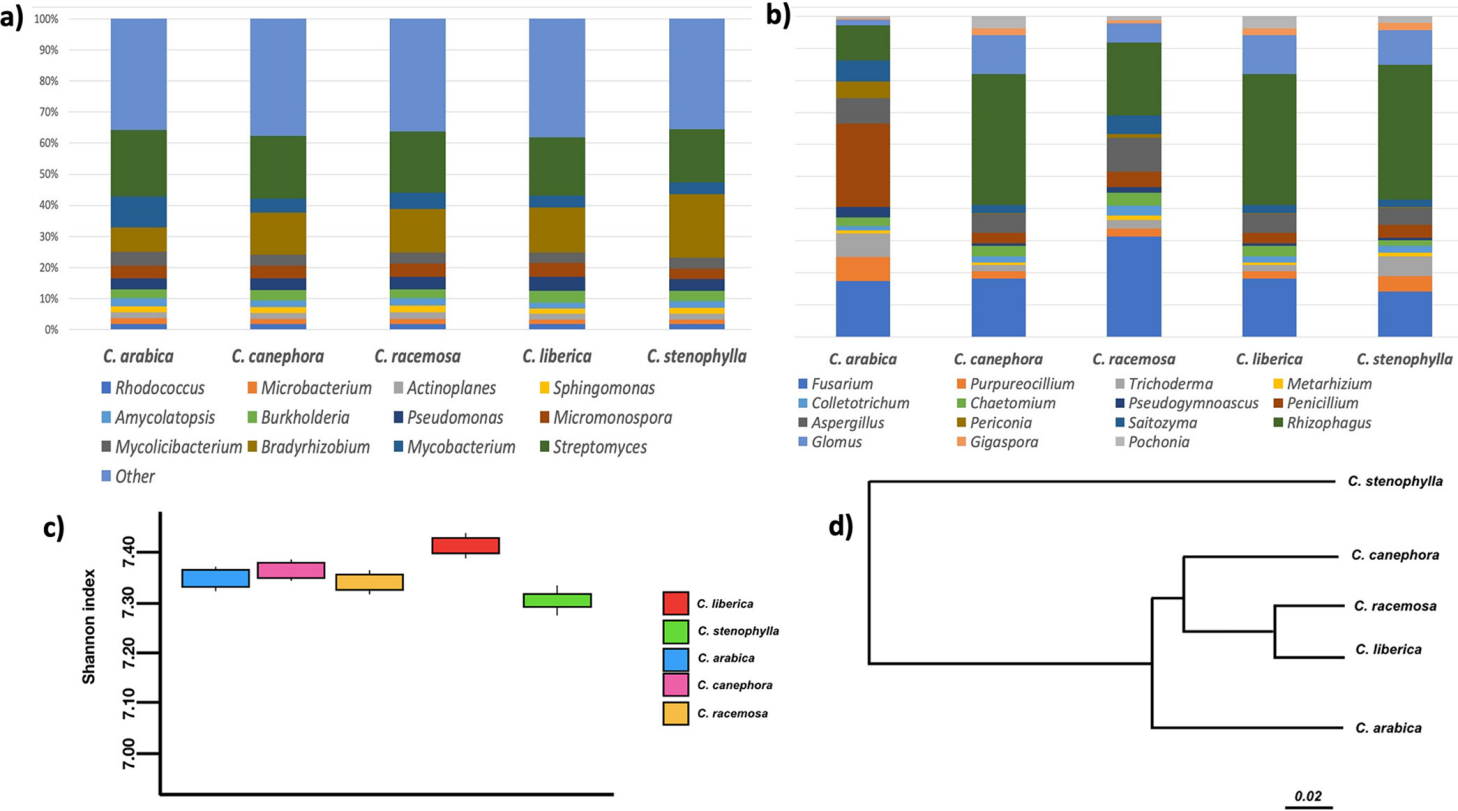
(a, b) Relative abundances of bacterial (a) and fungal (b) genera from deep shotgun metagenomic sequencing of five different *Coffea* species. (c) Alpha diversity comparison of *Coffea* rhizospheres based on the Shannon index. The statistical analysis was done between all five communities using the Kruskal-Wallis test (*P* > 0.01). (d) Weighted UniFrac-based cluster analysis of microbial community compositions among different *Coffea* species.

**FIG 2:**
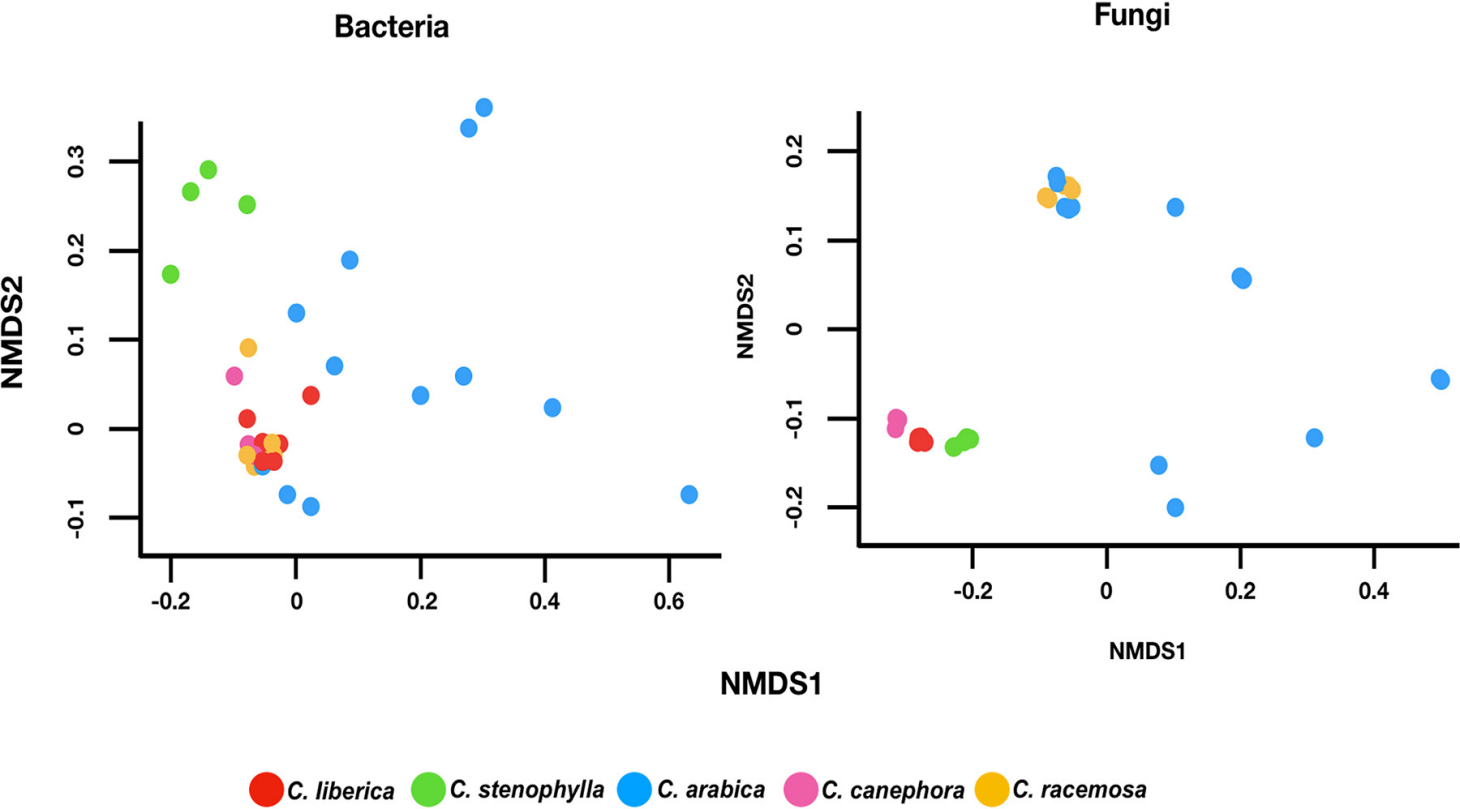
Nonmetric multidimensional scaling (NMDS) plots of microbial communities based on dissimilarities calculated using the Bray-Curtis indices of bacterial (stress = 0.04) and fungal (stress = 0.02) communities’ compositions.

We investigated the possible differences between the microbiomes by separating the analysis into bacteriomes and mycobiomes. The Kruskal-Wallis test revealed significant differences between the microbiomes both in the case of the bacteriomes (*P* = 4.026E−05) and for the mycobiomes (*P* = 0.03931). Dunn’s *post hoc* test revealed (Tables S3 and S4) that the bacteriome of C. stenophylla is statistically different from the rest, while the bacteriome of C. arabica also differs from all except that of C. canephora (*P* = 0.4955). The C. stenophylla mycobiome did not differ statistically from any other, whereas the C. arabica mycobiome differed from all except that of C. stenophylla (*P* = 0.1096). These data suggest that, taxonomically, the microbiomes of C. stenophylla and C. arabica diverge more from the others, which was verified in the weighted UniFrac-based cluster analysis (Fig. 1d) and NMDS (Fig. 2). Regarding taxonomic distribution, bacteria of the genera Streptomyces, Bradyrhizobium, and Mycobacterium (Fig. 1a) and fungi of the genera Fusarium, Trichoderma, Aspergillum, and Rhizophagus (Fig. 1b) are the prevalent ones. Streptomyces was the most prevalent bacterial group for all coffee trees, with the exception of C. stenophylla, where Bradyrhizobium dominated. About 26% of the detected Streptomyces sequences were not classified in any known species, which opens an opportunity to better explore this unknown potential. Several members of the bacteriomes here present are known as growth promoters, such as Bradyrhizobium, Pseudomonas, Burkholderia, Sphingomonas, Methylobacterium, Bacillus, and Azospirillum (25). For fungi, Metarhizium, Fusarium, Aspergillum, Colletotrichum, and Chaetomium were found more in C. racemosa, Penicillium and Periconia were found more in C. arabica, and mycorrhizal fungi Glomus and Rhizophagus in C. canephora, C. liberica, and mainly in C. stenophylla. Together, Glomus, Gigaspora, and Rhizophagus accounted for 36% of all of the fungi found. Other fungi with potential to promote plant growth were found, such as Aspergillus, Penicillium, and Trichoderma, these having reputed biocontrol action against plant pathogens (26). The Shannon’s diversity indices ranged from 7.30 to 7.40 (Fig. 1c).

### Functional traits.

**FIG 3:**
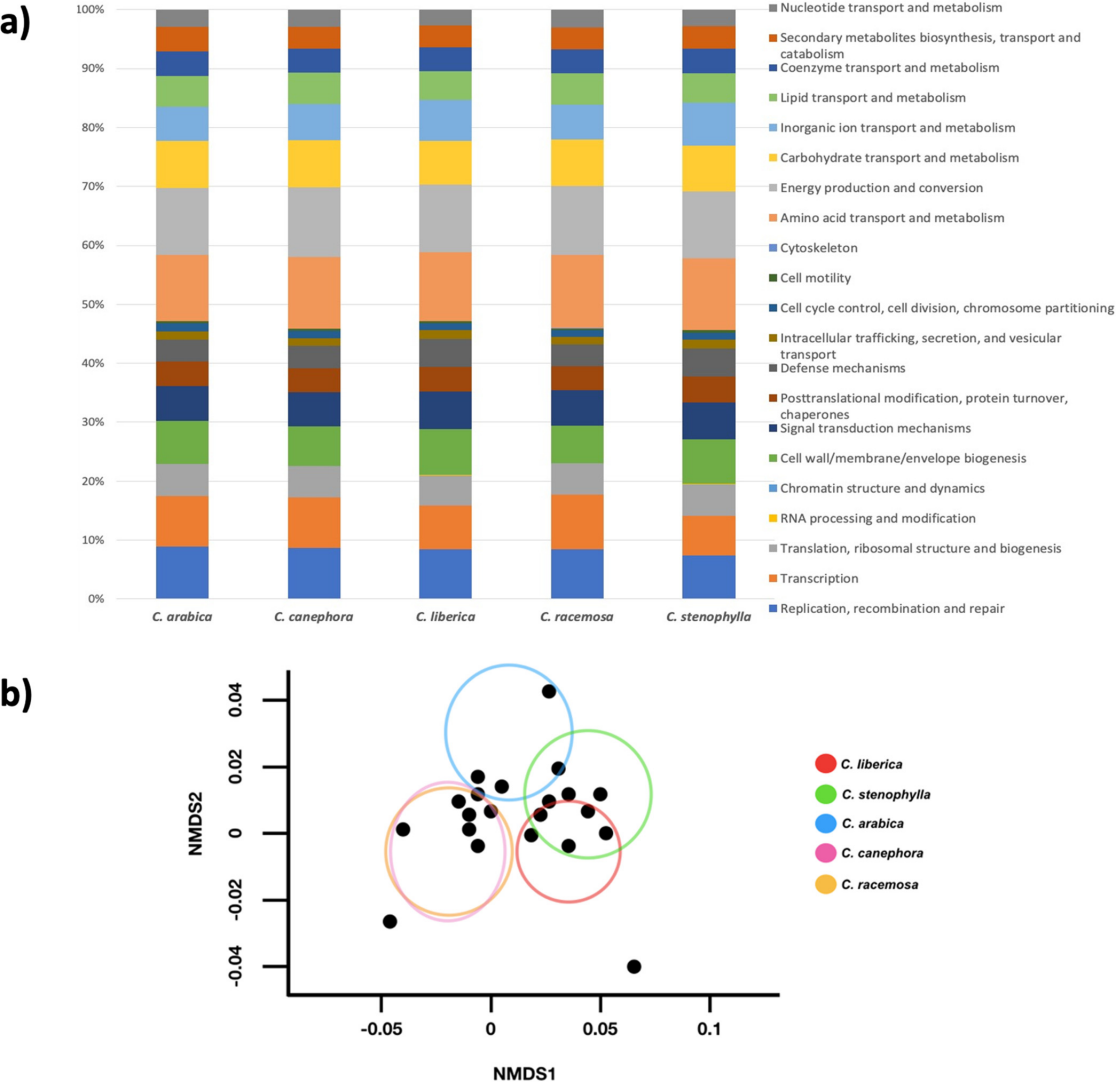
(a) Relative abundances of metagenes in the rhizosphere microbiome. (b) Nonmetric multidimensional scaling (NMDS) plot of microbial communities based on dissimilarities calculated using the Bray-Curtis indices of functional features (stress = 0.048).

Functional annotations were obtained for 14 to 31% of the metagenes by PfamScan and EggNOG mapper. Replication/recombination/repair, energy production, and amino acid transport/metabolism were the prevalent subsystems (Fig. 3a). We found that, functionally, the microbiome communities were significantly different (Kruskal-Wallis test; *P* = 0.0365), whereas those of C. canephora and C. racemosa did not differ significantly from each other (Dunn’s *post hoc* test; *P* = 0.2896) and formed a cluster in the NMDS (Fig. 3b). The Pfam annotation showed a large predominance of transporters; for example, ABC transporters (PF00005, PF00497, PF00496, and PF00532), major facilitator superfamily (PF07690), and proteins encoded by the *AcrB/AcrD/AcrF* family (PF00873), a class of proteins important for numerous processes, such as antibiotic resistance and transport of amino acids and carbohydrates from root growth (27, 28). This finding was not surprising considering that Streptomyces was the most commonly found group and it is a genus that has a rich repertoire of transport proteins (29) that play an important role in nutrient uptake and substrate secretion. We also recorded a large amount of sigma factor 54 (PF04552), which is important for biofilm formation, antibiotic production, and regulation of the production of siderophores and plant hormones (30, 31). We found several proteins involved in stress response, such as glyoxalase (PF12681), ferretin (PF00210), peroxidase (PF00141), stress response protein (PF05532), catalase (PF00199), and superoxide dismutase (PF00081), which are more frequently detected in the phyllosphere (32). Proteins involved in nitrogen fixation were widely found (PF12196, PF04319, and PF00142), reflecting the large number of rhizobia (Bradyrhizobium, Rhizobium, and Mesorhizobium) and nonrhizobia (Azospirillum, Beijerinckia, and Frankia) detected. This suggested that there might be some degree of nitrogen fixation, especially from Azospirillum. A considerable number of photosynthesis-related proteins were detected; for example, RuBisCo (PF02788, PF18087, PF00016, and PF00101) and Calvin cycle (PF00936, PF00502, PF00485, PF00162, PF01116, PF01383, and PF00101) enzymes, which were assigned to *Nitrosomonadales*, *Bradyrhizobiaceae*, purple sulfur bacteria, and unclassified *Verrucomicrobi*a. Many of the annotated genes might benefit plants through their involvement in multiple processes that enhance their growth. For example, some genes are involved in phosphate solubilization (PF01011) and phosphate transport (PF00005). We also found genes for the synthesis of nitric oxide (PF02613 and PF02239) but not for salicylate and indoleacetic acid. On the other hand, we detected genes for salicylate hydroxylase from *Burkholderiaceae* (PF01494), an enzyme that degrades salicylate and is linked to the ability of microorganisms to colonize their hosts (33). Genes involved in the metabolism of benzoate and quinate, two secondary metabolites found in *Coffea* (34, 35), were found, especially in members of *Actinobacteria* and *Burkholderiaceae* (PF03594 and PF01494). Surprisingly, we did not detect genes related to the catabolism of xanthine (caffeine/theobromine) or chlorogenic acid.

### Abiotic and biotic interactions.

**FIG 4:**
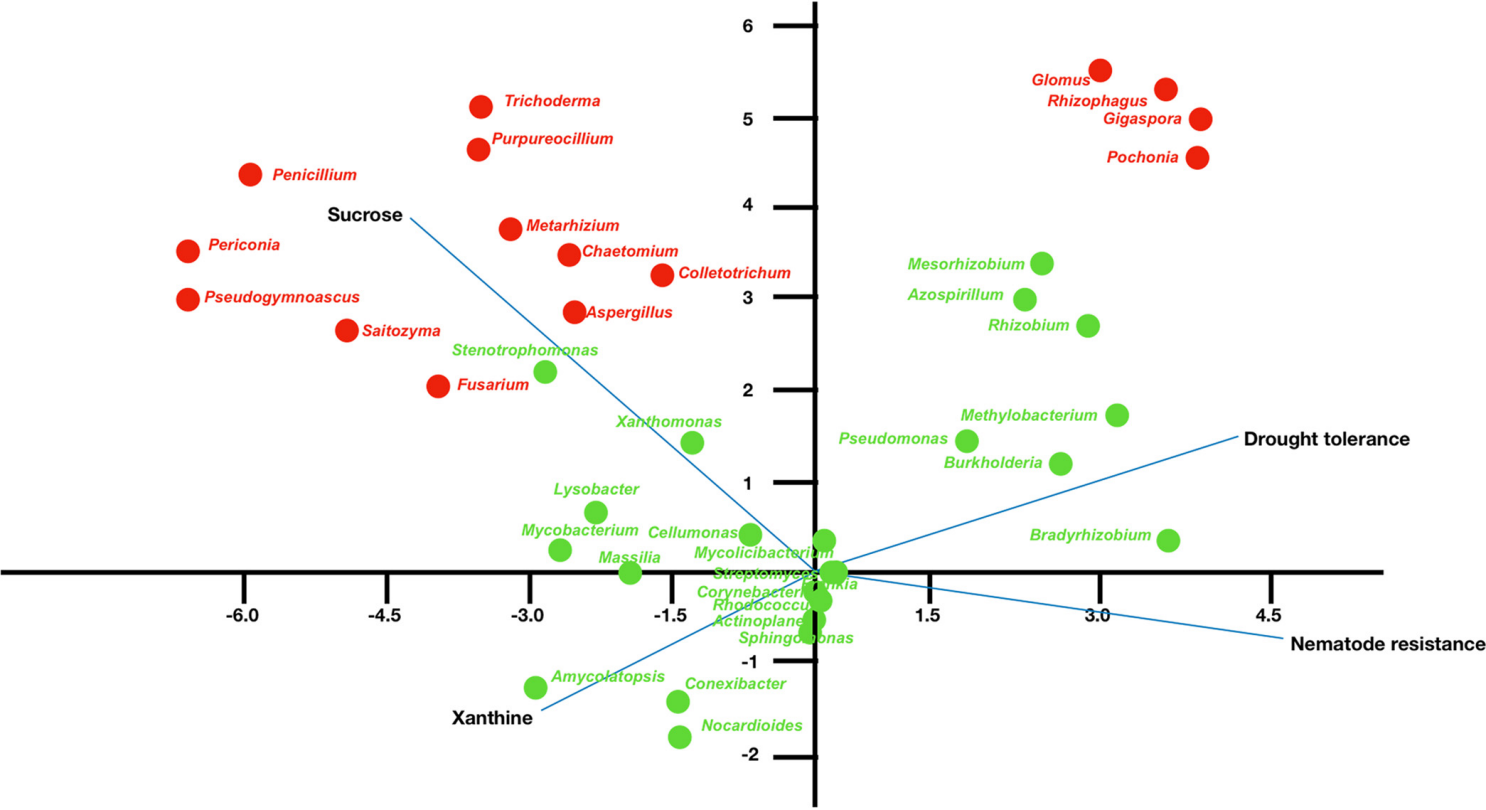
Canonical correspondence analysis of metagenomic sequence data and plant traits. Blue lines represent plant traits sucrose/xanthine concentration in rhizosphere, drought tolerance, and nematode resistance. Only genera with a relative abundance higher than 1% are used. Names in red are fungi, and names in green are bacteria.

Canonical correspondence analysis (Fig. 4) revealed that most fungi were impacted by the concentration of sucrose (Fig. S2) in the rhizosphere, while the concentration of xanthine seemed to impact few microbial groups (Amycolatopsis, Conexibacter, and Nocardioides). Interestingly, when we analyzed the relationships of microbiomes to the different tolerances of *Coffea* species to drought, we saw a correspondence between this characteristic and growth-promoting microorganisms like mycorrhizal fungi, Azospirillum, Pseudomonas, Methylobacterium, rhizobia, and Burkholderia. There was no correspondence between nematode resistance and any microbial group.

**FIG 5:**
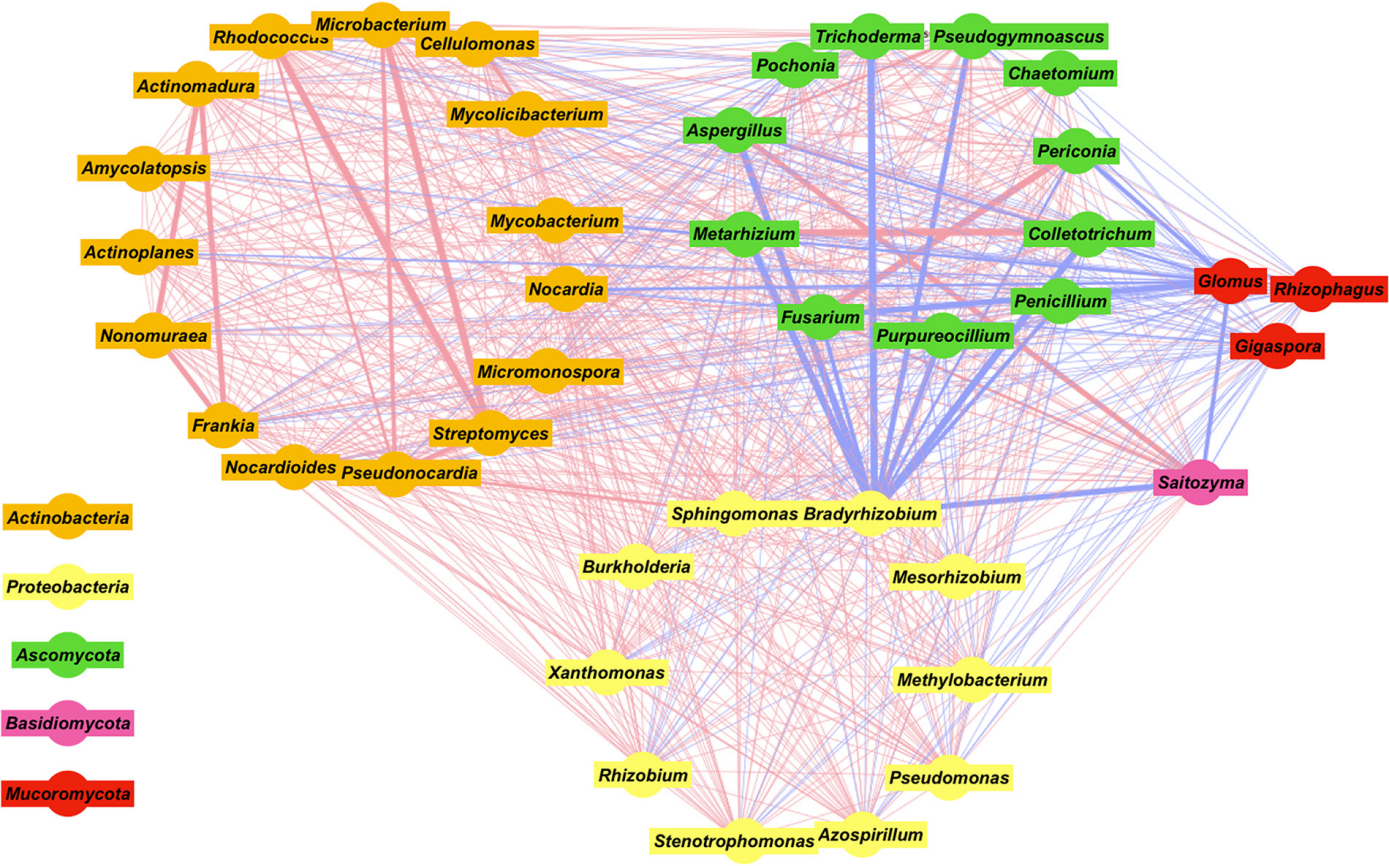
Network analysis from Cytoscape of bacterial and fungal communities, divided into color groups according to phyla. Blue lines represent negative correlations; red lines represent positive correlations. The thicker the line, the stronger the correlation.

The network diagram (Fig. 5) showed that Bradyrhizobium had intense negative correlations with at least 10 fungi; mycorrhizal fungi (Glomus, Gigaspora, and Rhizophagus) also had negative correlations with other microbes. On the other hand, actinobacteria had predominantly positive correlations among themselves, especially between the groups Actinomadura*-*Frankia*-*Nonomuraea and Rhodococcus*-*Microbacterium*-*Pseudonocardia*-*Streptomyces, where the correlation was more accentuated. The majority of the interactions were of a positive nature.

## DISCUSSION

In this work, we performed a study of the taxonomic and functional traits of the rhizosphere microbiomes of five *Coffea* species to better determine any possible differences. We expected that large differences would be detected between coffee trees, as the plant genotype has been identified as an important factor in the structuring of microbiomes (36–39). In fact, we saw that the microbiomes of C. arabica and C. stenophylla differed from those of C. racemosa, C. canephora, and C. liberica and exhibited different structures with respect to bacterial and fungal communities. We expected that the microbiomes of C. arabica and C. canephora would be more similar to each other, since C. arabica is the result of hybridization between C. canephora and Coffea eugenioides. This was true for the bacteriome but not for the mycobiome, which may suggest that C. arabica “inherited” the bacteriome from its parent, but not the mycobiome. We sought evidence to explain this pattern by verifying the possible effect of xanthine and sucrose on microbiome shaping. Apparently, sucrose had an influence on nonmycorrhizal fungi and some bacteria, especially Stenotrophomonas and Xanthomonas. Interestingly, xanthines had little impact on microbiomes. In fact, we did not detect metagenes for xanthine catabolism, which suggested that these compounds interfered little in microbiome modeling. Despite this, we do not discard the possibility of other secondary metabolites acting as microbiota modelers (40).

As seen in other reports (41–44), despite the great microbial diversity, a few species predominated, with *Proteobacteria* and *Actinobacteria* practically defining the rhizosphere of all *Coffea* species. Streptomyces, Mycobacterium, and Bradyrhizobium together made up 50% to 45% of the genera found. However, comparing our data with those found by Jurburg et al. (41) in Nicaraguan C. arabica, we saw differences between the microbiomes. In this work, *Actinobacteria* sequences were the fourth most detected. *Proteobacteria*, *Acidobacteria*, and *Verrucomicrobia* were the most prevalent and Acidobacteria and Verrucomicrobia were little detected in our work (Table S1a). These data suggest that environmental components, such as soil management, have important effects on the structures of *Coffea* microbiomes. In addition, Jurburg et al. (41) also found that older plants had microbiomes with less diversity, which may suggest microbial enrichment in the rhizosphere. Unlike their study, our plants are much older and have not been subjected to any type of management, so we speculate that the age of plants may significantly alter the microbiome at older ages. A comparison between plants with a greater variety of ages should be carried out to verify whether age actually influences the taxonomy of the microbiota. It can be speculated that different concentrations of certain minerals, such as potassium and calcium, in older plants may be involved in this phenomenon as well (41).

The prevalence of actinobacteria, especially Streptomyces, is a curious finding because this group is significantly underrepresented in the rhizospheres of other plants (38, 44–46). However, soils with large amounts of actinobacteria are usually suppressive for diseases, as the group is known to produce antimicrobial compounds (47–51). In fact, eutrophic environments, such as the rhizosphere, induce a greater negative interaction of actinobacteria with other microbes because the high nutrient levels increase their metabolism and the production of secondary metabolites, making them more aggressive colonizers (52). This prevalence may help explain the fact that the coffee tree has relatively few relevant soilborne diseases compared with other crops, especially in its native environment (53). As the most numerous group in coffee tree microbiomes, with the exception of C. stenophylla, we speculate that perhaps there is an intimate relationship between old *Coffea* plants and actinobacteria, as suggested by Seipke et al. (54), where the growth of the microorganism favors the host so much that this relationship becomes highly important. We also noted that there is a positive correlation within the actinobacteria (Fig. 5), which could denote some degree of cooperation or at least a direct noncompetition between the bacterial species. These assumptions are supported by the literature, where actinobacteria, when coinoculated, have higher growth than separated ones (55) and exhibit different strategies to compete for resources, avoiding direct competition (56). Considering the possible use of Streptomyces as a growth-promoting inoculant, the use of other actinobacteria as “collaborators” for the growth of Streptomyces should be taken into account.

A similar pattern was also observed in the fungal community. Mycorrhizal fungi, especially Rhizophagus, were widely found, but they dominated even more in C. stenophylla. It is interesting to note that C. stenophylla is more tolerant of drought and high temperatures than C. arabica (17). C. stenophylla grows in an open environment and is subject to greater insolation (17). The massive presence of mycorrhizal fungi may be related to these characteristics of C. stenophylla. Interestingly, we verified the correspondence between drought tolerance and mycorrhizal fungi, as well as growth-promoting bacteria such as Pseudomonas, Methylobacterium, Bradyrhizobium, and Azospirillum. In previous studies, these species have been linked to minimizing the effects of drought on their hosts (57–59); therefore, we suggest that the well-known drought tolerance of C. racemosa, C. stenophylla, and C. liberica (18, 60) may be due in part to these associations with beneficial microorganisms.

We also noticed that *Ascomycota* and *Basidiomycota* had a negative correlation with mycorrhizal fungi and Bradyrhizobium. The greater relative abundances of these two groups in C. canephora, C. stenophylla, and C. liberica coincided with the smaller abundances of *Ascomycota* and *Basidiomycota*, suggesting that mycorrhizal fungi and Bradyrhizobium may act as antagonists, especially for the control of Fusarium and Colletotrichum, which have been reported as potentially destructive pathogens for coffee trees (61). In fact, we detected in C. stenophylla many copies of gene sequences for citrate synthase (PF00285), an enzyme that participates in the production of citrates that act as siderophores in rhizobia. It is possible that this competition for iron can transform Bradyrhizobium into an antagonist of the fungi that colonize the rhizosphere. Aside from Streptomyces and Bradyrhizobium, we detected an abundance of Mycobacterium, which was also found in large amounts in tropical soils by Yeoh et al. (62). However, the ecology and function of Mycobacterium associated with roots remains unknown (62). It is suggested that the abundance of Mycobacterium in the rhizosphere is related to its ability to utilize simple sugars, such as fructose and glucose, molecules that are abundant in the rhizosphere (63). Numerous mycobacteria have been isolated in association with plants, some of them exhibiting beneficial effects on plant growth (64). Furthermore, these bacteria produce considerable amounts of bacteriocins (64), and thus, Mycobacterium can help to regulate the host’s bacteriome (65). These different interactions between microbiome and host, as well as within the microbiome, need further investigation to better understand how to best manipulate the benefits of this relationship.

Many of the functional traits found here were associated with microbe growth and survival in the rhizosphere (66). For example, plant roots release large amounts of carbohydrates, amino acids, and some secondary metabolites during their growth, and this release is used by the microbiota to colonize the rhizosphere (67, 68). We found a large number of transporters that may act in nutrient transport from the external environment to cells. With Streptomyces being the predominant genus, this finding is not surprising, because actinobacteria have large repertoires of transport proteins that are important mediators of complex processes like nutrient uptake, the concentration balance of elements, efflux of drugs/toxins, and secretion of proteins (69). We also found genes involved in the degradation of benzoate and quinate, compounds present in coffee tree tissues (70). It is possible that these two compounds are used as carbon sources by the microbiota. Furthermore, we detected the possible ability to catabolize salicylate, a hormone that mediates the immune response in plants and is essential to regulate the colonization of beneficial and pathogenic microorganisms, evading the host’s immune system (71, 72). Other characteristics found, such as phosphate solubilization, nitric oxide production, and nitrogen fixation, can greatly benefit the plant host by making available nitrogen and phosphorus and producing a hormone that is involved in the abiotic stress response. The presence of such characteristics can help guide the isolation of growth-promoting microorganisms, aiming at the development of a specific synthetic community for the coffee tree.

Although this study has promoted a taxonomic and functional analysis within the rhizosphere microbiomes of members of the genus *Coffea*, such studies are still at an early stage. We still do not know for sure the reason for the convergence of some microbiomes, nor whether it is found in plants under different edaphoclimatic conditions. An analysis with more plants vegetating under different conditions should be conducted. We think that, given the advanced age of the plants, their microbiomes must have undergone an intense enrichment that, in theory, may have selected a microbiome configuration that favors the hosts, but this needs to be investigated further as well. It has been found that continuous cultivation in a specific area leads a plant to recruit a consistent rhizosphere microbiome that can favor it (39, 73, 74). We suggest, then, that this could also be happening in the case of our coffee trees. It is possible that for each place of cultivation, there is a different “best configuration” for the microbiome, which would explain the differences between our findings and those of other authors (41).

### Conclusions.

In this work, we verified that genera such as Streptomyces, Mycobacterium, Bradyrhizobium, Burkholderia, Sphingomonas, Penicillium, Trichoderma, and Rhizophagus predominate in the rhizospheres of the five coffee species studied. We also saw that the xanthine content in the rhizosphere did not seem to influence the microbiota decisively, while the sucrose content mainly influenced the fungal population. Agronomic traits could also be influenced by the microbiota, where drought tolerance appeared to be linked to known growth-promoting microbes, while nematode resistance was not correlated with any particular group. The real effect of these microbes on these characteristics should be further investigated. Even so, we report a large number of microbes associated with members of the *Coffea* genus, many of them with a possible beneficial effect and which can work to improve coffee tree health and productivity.

## MATERIALS AND METHODS

### Experimental model and subject details.

Heathy plants, ranging from 54 to 77 years old, of Coffea arabica cv. Bourbon Vermelho, Coffea canephora, Coffea stenophylla, Coffea racemosa, and Coffea liberica var. *liberica* present in the Instituto Agronomico de Campinas, Sao Paulo, Brazil (22°53′S, 47°5′W, 664 m above sea level [a.s.l.]), were used for soil collection. For each species of *Coffea*, we selected four individuals (*n* = 4 for each *Coffea* species). Samples were collected 1 m from the tree trunk. All plants (*n* = 20 in total) were located on the same plot with clayey oxisol soil, pH 6.5, and under the same rainfall, temperature variation, and insolation conditions. The collections were made at the end of July (the “rest” period of the plants right after fruiting) and all on the same day. The top 5 cm of soil was removed, and fine roots (approximately 1 mm in diameter) from a depth of 5 to 20 cm were collected. The roots were shaken strongly to remove the attached soil, which was deposited in a 50-mL Falcon tube. This soil was stored at 4°C until DNA extraction on the same day.

### DNA extraction and sequencing.

DNA was extracted using a Mo Bio PowerSoil DNA extraction kit (Mo Bio Laboratories, Inc., Carlsbad, CA, USA) following the manufacturer’s instructions. The DNA quality and quantity were determined by using a NanoDrop device (Thermo Scientific, Wilmington, DE). Libraries were prepared using the Illumina Nextera DNA sample prep kit, with 50 ng of DNA input in each sample. The libraries were prepared using the NEBNext Ultra DNA library prep kit for Illumina. Paired-end sequencing was performed by NextSeq with the NextSeq 500/550 mid-output kit version 2 (300 cycles) at a read length of 150 bp.

### Metagenomic data analysis.

All bioinformatics analysis was performed by command line (R language) and supported by OmicsBox version 1.1 software (BioBam, Valencia, Spain). The clean reads from raw data were generated by removing adaptor sequences, trimming, and removing low-quality reads (reads with N bases and a minimum quality threshold of 20) using, respectively, the Cutadapt, Trimmomatic, and FastQC programs (75–77). The trimmed reads were mapped to the C. arabica genome using Bowtie2 software to identify and remove the *Coffea* host-originated reads (78). The pooled metagenomic reads from each *Coffea* species were assembled using metaSPAdes (79). The metagenes were predicted using Prodigal (80). For taxonomic information of the metagenes, taxonomic sequence classifier Kraken2 (81) was used. For functional information, PfamScan (which is used to search a protein sequence against a library of Pfam hidden Markov models [HMMs]) and eggNOG mapper (which is used to search a protein sequence against the eggNOG public database) tools from OmicsBox were used in the default mode. Canonical correlation analysis was performed to graphically represent whether the plant traits correlated with the microbial community structures, using the Past 4.02 program according to de Souza and Procópio (82). Also, UniFrac-based weighted cluster analysis was calculated using the Past 4.02 program (83) in order to build a *Coffea* phylogenetic tree based on its microbiomes.

### Network diagram.

The relationships of organisms sharing the same environment have been characterized generally by generating cooccurrence networks (84). In theory, positive pairwise correlations suggest interactions like symbiosis, mutualism, and commensalism, whereas negative pairwise correlations suggest competition, mutual exclusion, or parasitism. For the construction of the network diagram, we used the Pearson correlation index. For this, we used the CorrelationCalculator 1.0.1 program to normalize the data and calculate the indices. Then, through the index table, we used the Cytoscape 3.9.0 program to build the network diagram (85). To reduce complexity, only genera with a relative abundance of more than 1% were used.

### Statistical analysis.

The normality of the raw data was evaluated with the Shapiro-Wilk test (α = 0.05). As we verified that our data did not follow a normal distribution, we used the nonparametric Kruskal-Wallis test and Dunn’s *post hoc* test (α = 0.05) to determine if there were significant differences in alpha diversity across *Coffea* species. The taxonomic and functional dissimilarity analyses between *Coffea* microbiomes were performed based on nonmetric multidimensional scaling (NMDS) with the Bray-Curtis distance metric (beta diversity) using the VEGAN package in R software.

### Xanthine and sucrose measurement.

We decided to measure the concentrations of metabolites secreted by the roots into the surrounding soil, including (i) sucrose, because it is a very abundant compound in the soil around the root and is one of the triggers for the colonization of the rhizosphere for microbes (86), and (ii) the xanthine group, a group of alkaloids whose main members are caffeine and theobromine and which are widely produced by coffee plants (87). For the extraction and quantification of xanthine, we used the protocol of Huck (88) with changes. An amount of 0.2 mg of the rhizosphere soil collected for metagenomics was extracted with 4 mL 0.1 N HCl. After centrifugation (4 min at 3,000 × *g*), the precipitate was washed twice with 0.1 N HCl. The supernatants were neutralized (NaOH) and lyophilized. The residue was dissolved in 4 mL 0.1 N HCl and applied to Chromabond XTR SPE cartridges (Sorbent Technologies, Inc., GA, USA). After the liquid had passed through the cartridge, the xanthines were eluted with 5 mL of CHCl_3_-ethyl alcohol (EtOH) (95:5). The organic phase was evaporated, and the residue was dissolved in 1 mL methanol. This liquid was used for high-performance liquid chromatography (HPLC) using methanol (MeOH)/water/tetrahydrofuran (15:84:1). The UV absorbance at 280 nm was recorded for detection. Standard curves were made with caffeine and theobromine dissolved in MeOH/H_2_O (4:6). Sucrose was extracted using the protocol described by Ky et al. (89) with modifications. Soil samples were homogenized with distilled water and then heated to 60°C for 15 min. Colloidal material was precipitated with two solutions of zinc acetate and potassium hexacyanoferrate. The solution was filtered (0.1-μm pore diameter) and also analyzed using anion exchange chromatography.

### Nematodes and drought tolerances.

The characterization of tolerance or not to nematodes and drought was based on the observations of other authors (Aribi et al. [90] for nematode tolerance and Davis et al. [18], Mauri et al. [91], and Mishra [92] for drought tolerance).

### Data availability.

The raw sequencing reads were deposited in the NCBI BioProject database under accession number PRJNA793759. Other data supporting the findings of the study are available in this article and its supplemental material or from the corresponding author upon request.

## Supplementary Material

Reviewer comments
