# Supplementary material for: The Rhizosphere Microbiomes of Five Species of Coffee Trees
Source: Microbiol Spectr. 2022 Mar 15;10(2):e00444-22. doi: 10.1128/spectrum.00444-22 (PMC9045209; doi:10.1128/spectrum.00444-22)
Supplement: SUPPLEMENTAL FILE 1 — Supplemental material. Download SPECTRUM00444-22_Supp_1_seq4.pdf, PDF file, 3.9 MB [file spectrum00444-22_supp_1_seq4.pdf]

Supplementary Information

The rhizosphere microbiome of five species of coffee trees

de Sousa *et al.*

List of content

Supplementary information
Supplementary figures
Supplementary tables

Supplementary information

1. General information about *Coffea* species used in this work

Coffea arabica *L.*

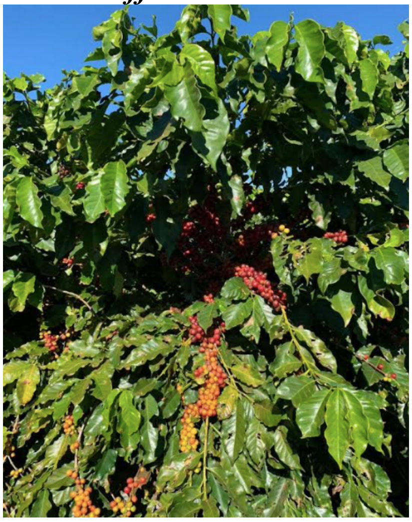

Planta age: 67 years old

Description: In the wild, plants can grow between 9 and 12 m. Commercially, cultivars grow between 2 and 5 m to facilitate harvesting.

Distribution: south-west Ethiopia, south-east Sudan and Kenya.

Ecology: Humid, evergreen forest; 950–1950 m; tolerant to low temperatures, but not frost;

Notes: natural hybrid between *C. canephora* and *C. eugenioides,* hybridization occurred between 1.08 million and 543,000 years ago; provides about 60% of the of the world's marketable coffee. Susceptive to nematodes and drought.

Coffea canephora *Pierre ex A.Froehner*

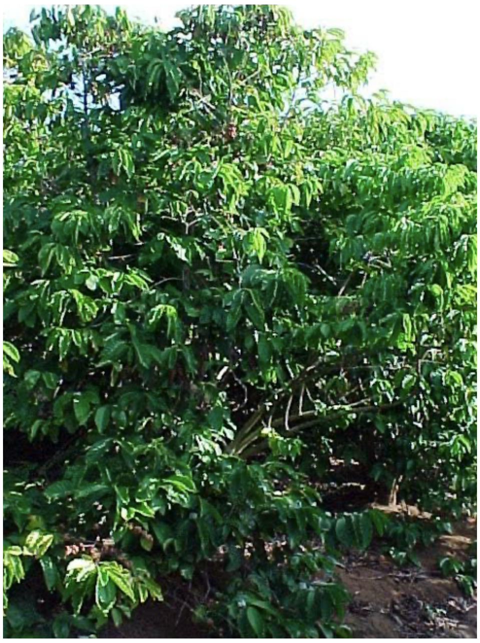

Plant age: 55 years old

Description: The plant grows as a robust tree or shrub to about 10 m tall.

Distribution: West Tropical Africa, west-central Tropical Africa, north-east Tropical Africa, east Tropical Africa, south Tropical Africa.
Ecology: Humid, evergreen forest; 50–1500 m.
Notes: contains more caffeine (2.7%) compared to *C. arabica* (1.5%); As it is less susceptible to pests and disease; provides about 40% of the of the world's marketable coffee. Susceptive to drought.

Coffea liberica *Bull. ex Hiern*

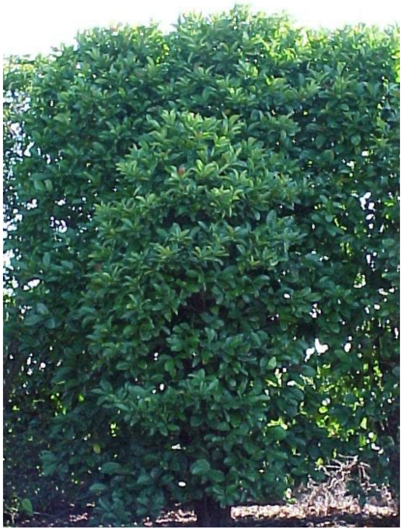

Plant age: 77 years old
Description: trees reaching up to 20 m high.
Distribution: West Tropical Africa, north-east Tropical Africa, west-central Tropical Africa and north-east Tropical Africa.
Ecology: Humid, evergreen forest; 80–1800 m
Notes: contains less caffeine (1,23%) than *C. canephora* and *C. arabica*; provides less than 1% of the world's marketable coffee. Tolerant to nematodes. Resistant to drought.

Coffea racemosa *Lour.*

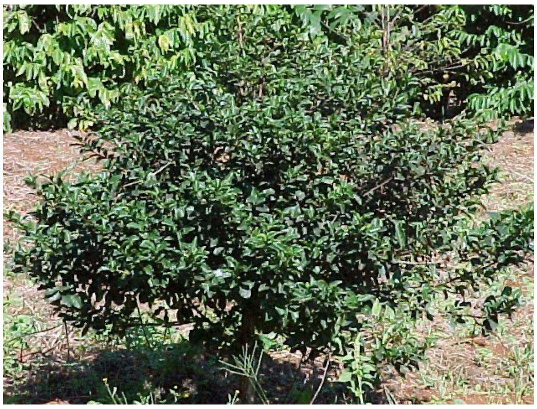

Plant age: 54 years old
Description: shrub or small tree growing up to 3.5 m tall.
Distribution: Southern Tropical Africa, southern Africa, western Indian Ocean.
Ecology: Seasonally dry, littoral forest; 0–600 m.
Notes: less than half of caffeine found in *C. arabica*. Tolerant to drought.

Coffea stenophylla *G.Don*

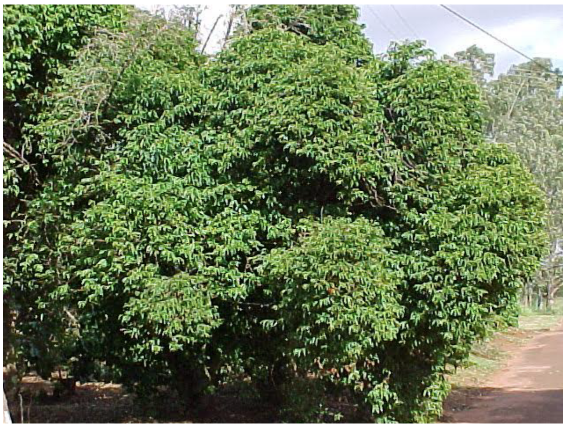

Plant age: 55 years old
Description: shrub or tree with 6 m tall.
Distribution: West Tropical Africa.
Ecology: generally restricted to drier areas; 200 m.
Notes: resistant to rust, nematodes, drought and hot temperatures.

Supplementary figures

S1. Taxonomic distribution at phylum level a) Bacteria b) Fungi

a)

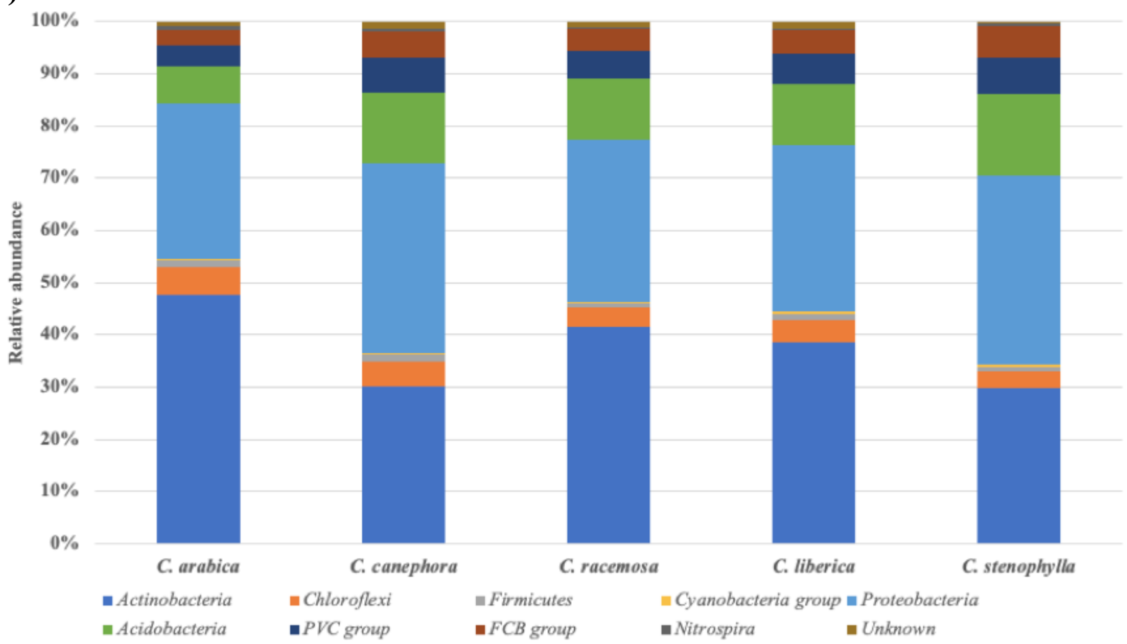

b)

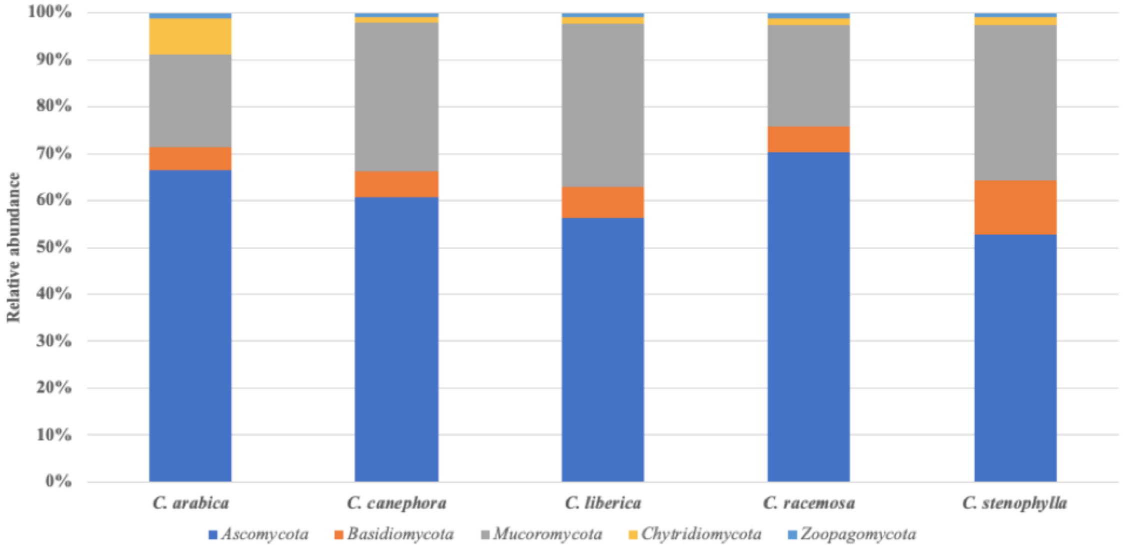

S2. Concentration of sucrose and xanthine in present in the rhizospheric soil of the five coffee trees. *t-test revealed significant differences p<0.05

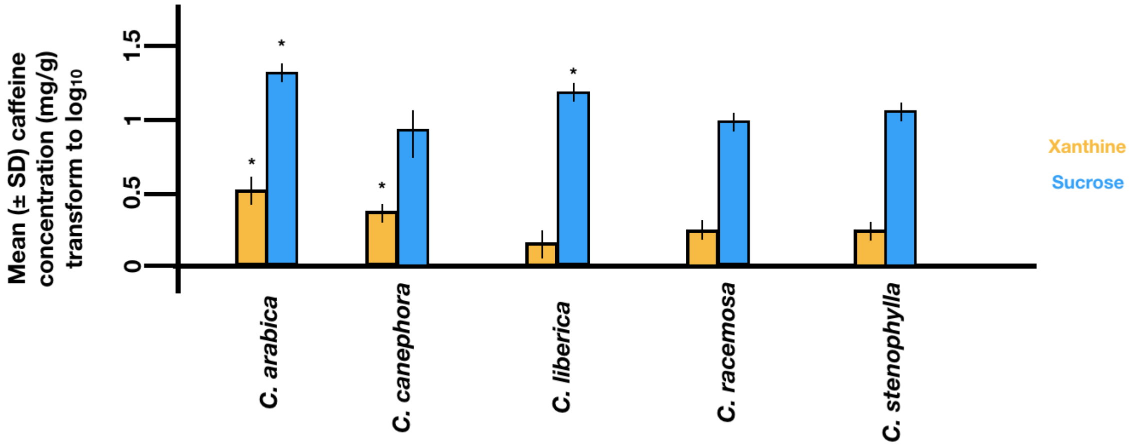

Supplementary tables

Table S1. Sequencing and assembly information

	C. arabica	*C. canephora*	*C. liberica*	*C. racemosa*	*C. stenophylla*
**Number of contigs**	307.084	220.709	337.161	152.825	325.530
**Largest contig**	16.528	30.385	71.539	20.424	40.621
**Total length**	23.933.224	8.716.857	112.414.477	3.181.133	88.946.051
**GC (%)**	64.29	67.78	61.8	67.29	59.51
**N50**	814	779	1.001	638	620
**L50**	7.936	3.525	7.276	1.804	3.405
**Number of genes**	333.699	233.961	368.295	159.124	343.674
**Minimal length**	60	60	60	60	56
**Maximum length**	6.306	3.630	7.440	4.581	4.062
**Average length**	263.99	239.42	268.9	230.88	270.25

Table S2. Sequence distribution (%) between classified, unclassified and between different kingdoms

	**Unclassified**	**Classified**	**Celular organisms**	**Virus**	**Bacteria**	**Archaea**	**Eukaryota**
C. arabica	59	41	99.98	0.02	98	0.5	1
C. stenophylla	60	40	99.97	0.03	99	0.6	0.4
C. liberica	60	40	99.98	0.02	99	0.5	0.5
C. racemosa	59	41	99.97	0.03	99	0,3	0.7
C. canephora	60	40	99.92	0.02	99	0.5	0.5

Table S3. Dunn's post hoc test for the bacteriome generated by Past4. Significantly values in colored ($p<0.05$)

	C. arabica	*C. canephora*	*C. racemosa*	*C. liberica*	*C. stenophylla*
C. arabica		0.4955	0.02175	0.0489	3.55E-06
C. canephora	0.4955		0.1067	0.1977	7.665E-05
C. racemosa	0.02175	0.1067		0.7451	0.01921
C. liberica	0.0489	0.1977	0.7451		0.007661
C. stenophylla	3.55E-06	7.665-05	0.01921	0.007661	

Table S4. Dunn's post hoc test for the mycobiome generated by Past4. Significantly values in colored ($p<0.05$)

	C. arabica	*C. canephora*	*C. racemosa*	*C. liberica*	*C. stenophylla*
C. arabica		0.01442	0.005693	0.01442	0.1095
C. canephora	0.01442		0.7502	1	0.3974
C. racemosa	0.005693	0.7502		0.7502	0.2442
C. liberica	0.01442	1	0.7502		0.3972
C. stenophylla	0.1095	0.3974	0.2442	0.3974	
